# The effect of cold-knife conization on pregnancy outcomes in patients with cervical lesions

**DOI:** 10.1371/journal.pone.0278505

**Published:** 2022-12-01

**Authors:** Yue Gao, Huali Wang, Yunyun Xiao

**Affiliations:** Department of Gynecology, Dalian Women and Children’s Medical Group, Dalian, Liaoning, People’s Republic of China; University of Palermo: Universita degli Studi di Palermo, ITALY

## Abstract

**Objective:**

To analyze the pregnancy outcomes of patients with cervical lesions treated by cold-knife conization (CKC).

**Methods:**

Clinical data of healthy pregnant women and pregnant women who underwent CKC in Dalian Women and Children’s Medical Group from March 2010 to December 2019 were retrospectively analyzed. These patients were divided into a CKC group and a control group according to inclusion and exclusion criteria. Statistical methods were used to compare pregnancy and delivery outcomes between the two groups.

**Results:**

There were 400 patients in CKC group and control group, with 200 patients in each. There was no significant difference in the mode of delivery, abortion, ectopic pregnancy, in-hospital perinatal management, and cervical cerclage between the CKC group and the control group (*P*>0.05). The rates of preterm delivery, premature rupture of membranes, cesarean section, and neonatal admission in the CKC group were higher than those in the control group (*P*<0.05). In the CKC group, the incidence of premature rupture of membranes within six months postoperatively was higher than that after six months (*P*<0.05). The incidences of preterm delivery and premature rupture of membranes were not completely consistent in different conization ranges (*P*<0.05).

**Conclusion:**

CKC increases the incidence of preterm delivery, premature rupture of membranes, and neonatal adverse outcomes. Conization height can predict the occurrence of preterm delivery. Delaying pregnancy after surgery can reduce the incidence of adverse outcomes during the perinatal period.

## Introduction

Cervical cancer is one of the most common cancers affecting the health of women all over the world. As a precancerous lesion of cervical cancer, cervical squamous intraepithelial lesion (SIL) has an increasing incidence and a younger trend [1]. At the same time, the postponement of childbearing in women increases the demand for patients to retain reproductive function, so considering both tumor outcome and reproductive outcome has become an important issue in treatment.

According to the 2019 American Society of Colposcopy and Cervical Pathology (ASCCP) guidelines, women diagnosed with high-grade squamous intraepithelial lesions (HSIL) cervical intraepithelial neoplasia grade 2 (CIN2) and less than 25 years old or ≥ 25 years of age who wish to have children can be temporarily monitored, but if CIN2 persists for two years or progresses to cervical intraepithelial neoplasia grade 3 (CIN3), a cervical conization is required [2]. Cervical conization, comprising cold knife conization (CKC) and annular loopelectrical excision technique, was presently the standard surgery for the aforementioned indications [3,4]. To avoid missing early or latent cervical cancer, the objective of the therapy was to thoroughly eradicate the lesion and prevent its transformation into cancer. Previous studies identified the women may suffer from impaired emotional well-being and reduced quality of life [5] even in early stage of cervical cancer and the fertility is also damaged by fertility-sparing surgery [6] or cervical conization [7].

With the trend of cervical lesion rejuvenation, more and more patients undergoing cervical conization desire fertility, and whether cervical conization has an impact on patients’ fertility, mode of delivery, and pregnancy outcomes has not yet been definitively concluded. CKC is a surgical treatment used to detect or treat cervical dysplasia. A cone-shaped piece of the cervix is excised in order to eliminate a cervical lesion and the complete transformation zone. Practitioners might employ this method when a pap smear and biopsy specimen contradict each other. It may be employed if histological findings are much less severe than cytology results, if there is evidence of severe dysplasia, or even if stage 1A squamous cell cervical carcinoma is present. Unlike other cervical conization methods, few studies have reported on the impact of CKC on fertility [7,8]. Thus, this retrospective study aimed to understand the impact of CKC on pregnancy outcomes. The second aim is to analyze the possible factors influencing these outcomes so as to provide reasonable management recommendations for patients with cervical lesions with fertility requirements.

## Materials and methods

### Research data

This study was a retrospective case-control study. The study was approved by the Dalian Maternal and Child Health Hospital Ethics Committee. The patients who underwent CKC and got pregnant after CKC in the Dalian Women and Children’s Medical Group from March 2010 to December 2019 were enrolled as the CKC group.

The enrollment criteria were as follows: (1) Women who underwent CKC in our hospital, (2) the postoperative pathological identified they had cervical HSIL, cervical squamous cell carcinoma stage Ia1 or AIS, (3) no recurrence during postoperative follow-up; (4) age between 25–35 years old; (5)without history of bad obstetric histories or pregnancy contraindications; (6) get pregnant after surgery.

Exclusion criteria: (1) women with the pathological classification higher than stage Ia1, (2) those with postoperative pathological upgrade and recurrence after CKC (including lesions with positive margins); (3) women with a history of infertility or known factors that could lead to infertility; (4) women with clear contraindications for natural delivery before pregnancy; (5) Women with other major diseases.

In accordance with the premise of matching age, pregnancy, and delivery order, the same number of pregnant women in the CKC group who experienced normal labor in our hospital over the same period were picked at random and designated as the control group.

### Observation parameters

The baseline characteristics, including age, history of pregnancy, and history of smoking, were collected and analyzed between the two groups. To understand the impact of CKC on pregnancy, the following parameters were collected and compared: mode of conception, abortion rate, ectopic pregnancy rate, hospitalized miscarriage rate during pregnancy, cervical cerclage rate, premature birth rate, mean gestational age, premature rupture of membranes rate, and premature rupture of membranes mean gestational age. Subgroup analyses were conducted to explore the factors associated with bad pregnancy outcomes. The postoperative pregnancy intervals, cone heights, cone floor transverse diameters, and cone volume were set as subgroups. Moreover, the optimal cutoff levels for cone heights and volume were measured, and the abilities of these cutoff levels to predict preterm delivery were also analyzed.

### Statistical methods

SPSS25.0 statistical software was used for data analysis. The measurement data, in accordance with the normal distribution, is expressed as the mean ± standard deviation (`*x* ± s). Counting data is described by frequency and constituent ratio. Measurement data in accordance with the normal distribution, satisfying the homogeneity of variance are analyzed by t-test and analysis of variance. A pairwise comparison is made by the SNK method. The t-test is used when the variance is not homogeneous. The χ^2^ test and Fisher’s exact test are used to compare the measurement data. The Spearman rank correlation is used for data that does not obey the bivariate normal distribution, and the receiver operator characteristic curve (ROC) curve and area under the curve (AUC) are used to measure the predictive value of cone height and volume for preterm delivery. Statistical significance was set at *P*<0.05.

## Results

### Baseline characteristics

A total of 400 patients were included in our study, with 200 patients in each group. The mean age was 31.23±2.57 in CKC group and 31.05±2.68 with no significant difference between the 2 groups (*P* = 0.494). Similarly, the gestation times (*P* = 0.107), parturition times (*P* = 0.916) and smoking history (*P* = 0.457) also had no significant difference between the two (Table 1). In the CKC group, there were 195 cases of cervical HSIL, 3 cases of stage A1 cervical squamous cell carcinoma, and 2 cases of AIS.

**Table 1:** Comparison of general data between CKC group and control group.

	CKC group, N = 200	Control group, N = 200	Test value	P value
**Age, years. Mean** (±SD)	31.23±2.57	31.05±2.68	*t* = 0.685	0.494
**Gestation times**, n/N (%)			*χ*^2^ = 4.468	0.107
0	95/200(47.5)	96/200(48.0)		
1	60/200(30.0)	74/200(37.0)		
≥2	45/200(22.5)	30/200(15.0)		
**Parturition times**, n/N (%)			*χ*^2^ = 0.333	0.916
0	157/200(78.5)	161/200(80.5)		
1	39/200(19.5)	35/200(17.5)		
2	4/200(2.0)	4/200(2.0)		
**Smoker**, n/N (%)	10/200(5.0)	7/200(3.5)	*χ*^2^ = 0.553	0.457

Cervical transformation zone and postoperative pathological results in CKC group.

Comparison of pregnancy outcomes between the CKC group and the control group.

There was no significant difference in mode of conception (*P* = 0.603), abortion rate (*P* = 0.292), ectopic pregnancy rate (*P* = 0.778), hospitalized miscarriage rate during pregnancy(*P* = 0.641), and cervical cerclage rate (*P* = 0.109) between the two groups.

The incidence of preterm delivery in the CKC group was higher than that in the control group (10.5% vs 4.5%, *P =* 0.023), but the mean gestational week at premature birth had no difference between the two groups (34.88±1.19 vs 33.30±2.74, *P* = 0.131). Moreover, the incidence of premature rupture of membranes was higher than that in the CKC group (27% vs 20.5%, *P* = 0.005). The mean gestational week at the time of premature membrane rupture was lower in the CKC group than in the control group (36.14±4.45 vs 37.72±4.77, *P* = 0.001) (Table 2).

**Table 2:** Comparison of mode of conception and adverse pregnancy outcomes between the CKC group and the control group.

	CKC group, N = 200	Control group, N = 200	Test value	P value
**Mode of conception**, n/N (%)			*χ*^2^ = 1.010	0.603
Natural conception	194/200(97.0)	196/200(98.0)		
Artificial insemination	3/200(1.5)	1/200(0.5)		
IVF-ET	3/200(1.5)	3/200(1.5)		
**Abortion**, n/N (%)	17/200(8.5)	10/200(5.0)	*χ*^2^ = 2.465	0.292
**Ectopic pregnancy**, n/N (%)	7/200(3.5)	6/200(3.0)	*χ*^2^ = 0.080	0.778
**Hospitalized miscarriage during pregnancy**, n/N (%)	25/200(12.5)	22/200(11.0)	*χ*^2^ = 0.217	0.641
**Cervical cerclage**, n/N (%)	8/200(4.0)	2/200(1.0)	*χ*^2^ = 2.564	0.109
**Premature birth**, n/N (%)	21/200(10.5)	9/200(4.5)	*χ*^2^ = 5.189	0.023
**Premature birth, Mean gestational week**(w±SD)	34.88±1.19	33.30±2.74	*t* = 1.658	0.131
**Premature rupture of membranes**, n/N (%)			*χ*^2^ = 10.531	0.005
None	146/200(73.0)	159/200(79.5)		
preterm	21/200(10.5)	5/200(2.5)		
full term	33/200(16.5)	36/200(18.0)		
**Premature rupture of membranes, mean gestational week**(w±SD)	36.14±4.45	37.72±4.77	*t* = 3.608	0.001

SD: Standard deviation.

#### Comparison of perinatal and neonatal outcomes between the two groups

The mean gestational week at delivery in the CKC group was lower than that in the control group (38.75±1.75 vs.39.29±1.76, *P* = 0.004), and the rate of cesarean section in the CKC group was higher than that in the control group (64/176, 36.4% vs. 49/184, 26.6%, *P* = 0.047). The fetal weight (3291.43±452.02g vs. 3360.49±499.15g, *P*>0.05) and the ratio of low-birth-weight infants (11/175, 6.3% vs. 4/183, 2.2%, *P*>0.05) had no difference between the two groups. The incidence of neonatal complications was significantly higher in the CKC group (15.4% vs 7.8%, *P* = 0.001).

### The impact of pregnancy intervals after CKC

The CKC group was distributed into subgroups according to different time intervals of pregnancy after CKC (≤6 month, 6–12 months, and >12 months). The results showed that there was no significant difference in the incidence of premature birth among the three subgroups (*P* = 0.932), but the incidences of premature rupture of membranes were significantly different (P = 0.026) (Table 3). The interval of postoperative pregnancy was negatively associated with the occurrence of premature rupture of membranes (r = -0.189, *P* = 0.007).

**Table 3:** The impact of postoperative pregnancy intervals (t) on preterm delivery and premature rupture of membranes.

	≤6 Month, N = 47	6 Month <t≤12 Month, N = 51	>12 Month, N = 102	Test value	P value
**Premature birth**, n/N (%)	5/47(10.6)	6/51(11.8)	10/102(9.8)	*χ*^2^ = 0.140	0.932
**Premature rupture of membranes**, n/N (%)	19/47(40.4)	15/51(29.4)	20/102(19.6)	*χ*^2^ = 7.276	0.026

### The impact of cervical conization size

The CKC group was again subdivided according to conization specimen size using the following parameters: height of cone (<15mm, 15-20mm, 20-25mm, >25mm), transverse diameter of cone bottom (<15mm, 15-20mm, 20-25mm, >25mm) and volume of cone (<2.0cm^3^, 2.0–3.0cm^3^, 3.0–4.0cm^3^, >4.cm^3^).

We identified that cone heights (r = 0.345 and 0.246, *P* = 0.015 and 0.034) and cone volumes (r = 0.276 and 0.198, *P* = 0.035 and 0.048) were positively associated with a higher rate of preterm delivery and premature rupture of membranes. Conversely, no correlation was identified among cone floor transverse diameters, preterm delivery, and premature rupture of membranes (Tables 4–6).

**Table 4:** The impact of cone heights on preterm delivery and premature rupture of membranes.

	<15mm, N = 11	15mm≤h<20mm, N = 54	20mm≤h<25mm, N = 99	≥25mm, N = 36	Test value	P value
**Premature birth**, n/N (%)	0/11(0.0)	3/54(5.6)	5/99(5.1)	13/36(36.1)	*χ*^2^ = 30.951	<0.001
**Premature rupture of membranes**, n/N (%)	0/11(0.0)	13/54(24.1)	23/99(23.2)	18/36(50.0)	*χ*^2^ = 14.678	0.002

**Table 5:** The impact of cone floor transverse diameters on preterm delivery and premature rupture of membranes.

	<15mm, N = 9	15mm≤d<20mm, N = 29	20mm≤d<25m, N = 95	≥25mm, N = 67	Test value	P value
**preterm delivery**, n/N (%)	0/9(0.0)	2/29(6.9)	8/95(8.4)	11/67(16.4)	*χ*^2^ = 4.390	0.222
**Premature rupture of membranes**, n/N (%)	2/9(22.2)	6/29(20.7)	26/95(27.4)	20/67(29.9)	*χ*^2^ = 0.973	0.808

**Table 6:** The impact of cone volumes on preterm delivery and premature rupture of membranes.

	<2.0cm^3^, N = 68	2.0cm^3^≤V<3.0cm^3^, N = 78	3.0cm^3^≤V<4.0cm^3^, N = 33	≥4.0cm^3^ n = 21	Test value	P value
**Premature birth**, n/N (%)	3/68(4.4)	8/78(10.3)	1/33(3.0)	9/21(42.9)	*χ*^2^ = 28.043	<0.001
**Premature rupture of membranes**, n/N (%)	12/68(17.6)	28/78(35.9)	5/33(15.2)	9/21(42.9)	*χ*^2^ = 11.180	0.011

**Fig 1:**
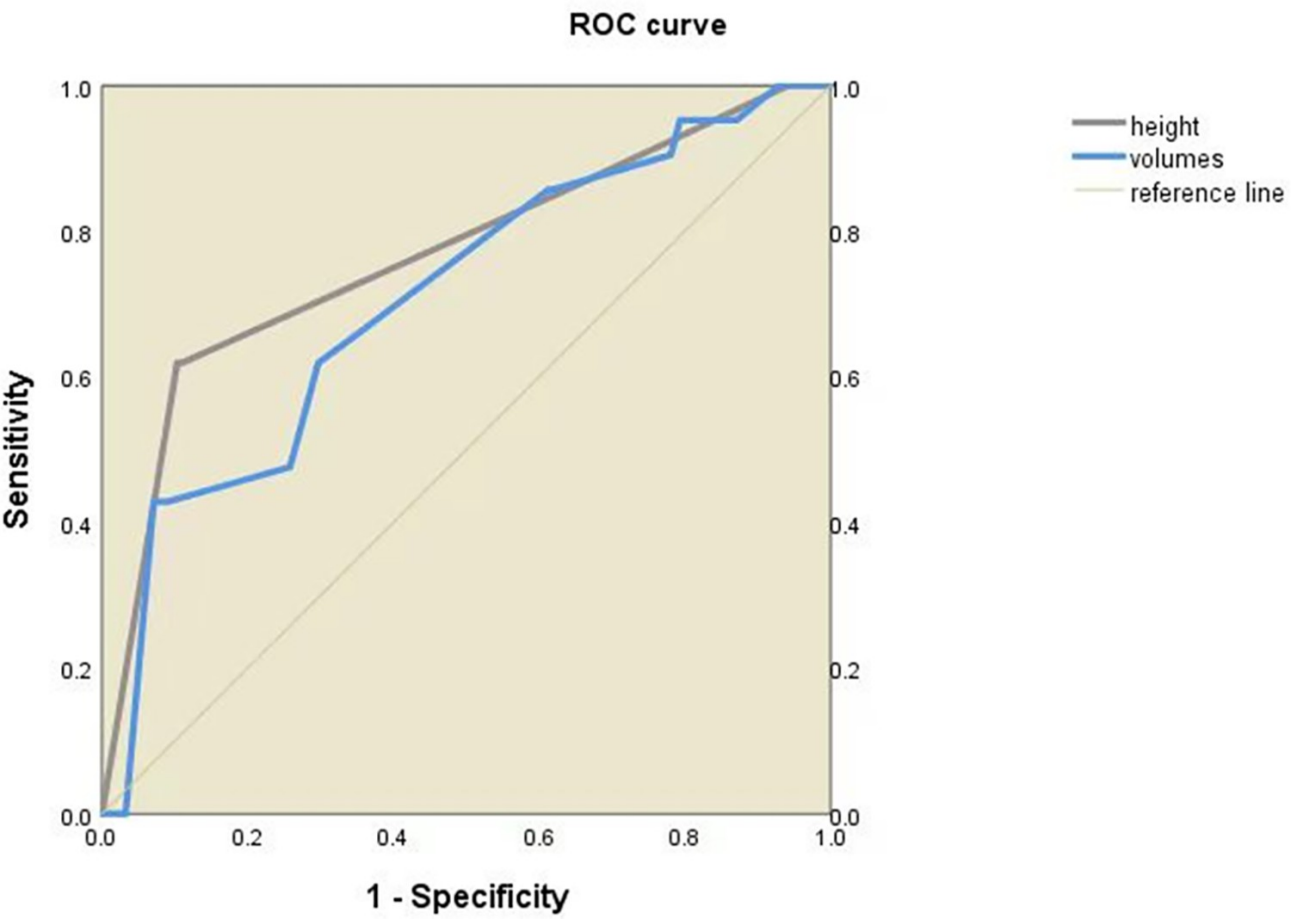
Preterm delivery is predicted using the ROC curve with cone height and volume.

**Fig 2:**
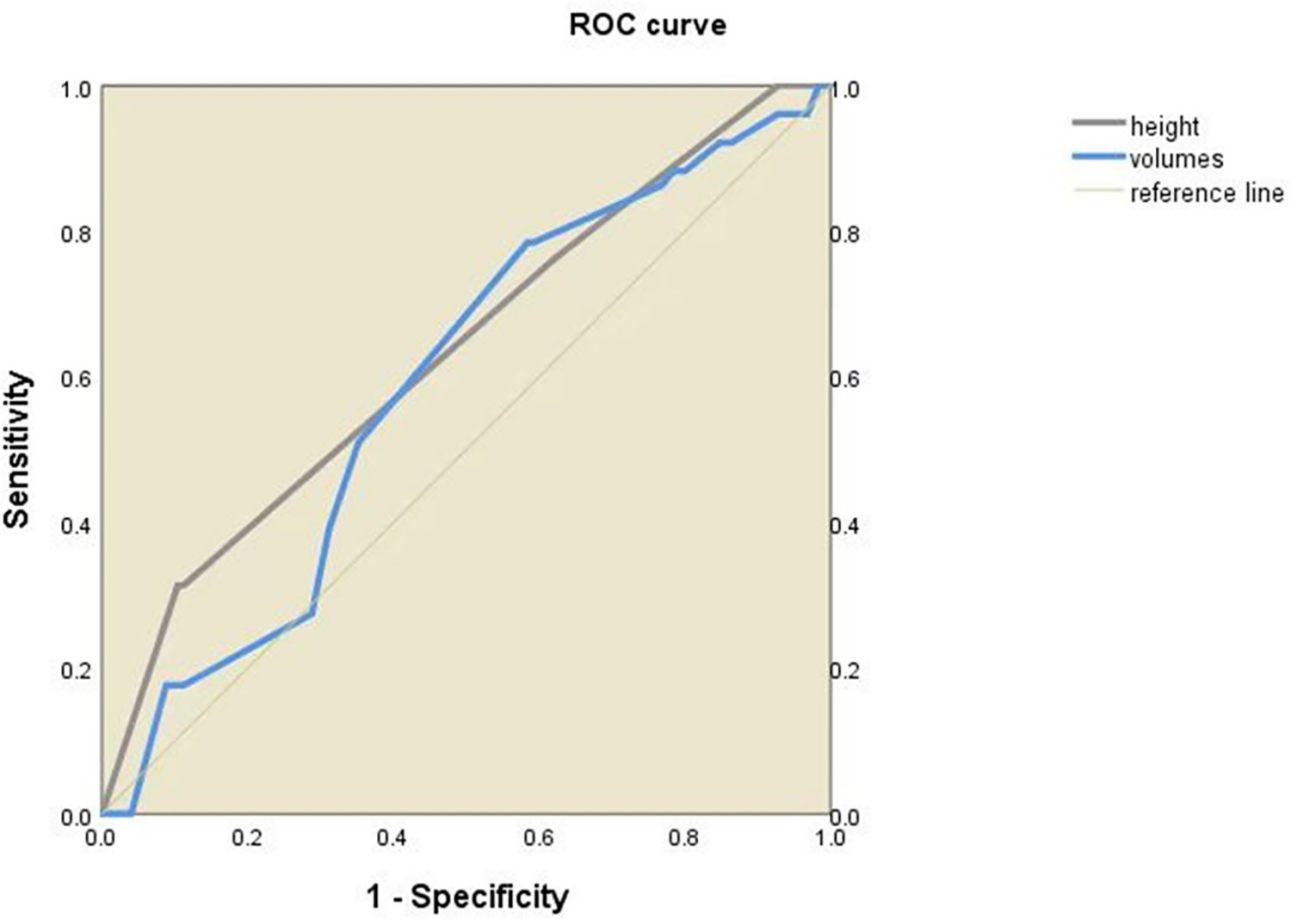
Premature rupture of membranes is predicted using the ROC curve with cone height and volume.

The ROC curve shows that both the cone height and the cone volume can be used to predict the occurrence of preterm delivery. The AUC was 0.766 (95% CI 0.643–0.889, *P*<0.001) and 0.708 (95% CI 0.587–0.829, *P*<0.05) for cone height and cone volume to predict preterm birth (Fig 1). Moreover, cone height was identified to have the ability to predict premature rupture of membranes (AUC = 0.635, 95% CI 0.544–0.727, *P*<0.05), but the ability of cone volume was not identified (AUC = 0.592, 95%CI 0.502–0.682, *P*>0.05) (Fig 2).

In order to further verify their predictive ability for preterm delivery, we used ROC curve analyses to determine the best cutoff level for cone height and volume, and the results were 2.4 cm and 4.0 cm^3^, respectively. The number of patients who had cone height and volume above the cutoff level was able to predict preterm delivery (*χ*^2^ = 35.754 and 23.481, *P*<0.001, and <0.001) (Table 7). Moreover, the accuracy and sensitivity for cone height and volume cutoff level to predict preterm delivery were 86.36%, 89.68% and 86.93%, 92.90%, respectively (Table 8). The cutoff level may be a useful tool to predict preterm delivery.

**Table 7:** Predictions for preterm delivery by cone height and volume.

		Taper cut height(h)cm		Taper volume(V)cm^3^		Total
		<2.4	≥2.4	<4.0	≥4.0	
**Preterm delivery**	**-**	159	18	163	13	155
	**+**	9	14	13	11	21
**P value**			<0.001			<0.001

**Table 8:** Evaluation of the prediction of preterm delivery by cone height and volume.

	Cone height	Cone volume
**Accuracy**	86.36%	86.93%
**Sensitivity**	89.68%	92.90%
**Specificity**	61.90%	42.86%
**Misdiagnosis rate**	38.10%	57.14%
**Missed diagnosis rate**	10.32%	7.10%
**Youden index**	52.58%	35.76%
**Positive likelihood ratio**	2.35	1.63
**Negative likelihood ratio**	0.1667	0.1656
**Positive predictive value**	94.56%	92.31%
**Negative predictive value**	44.83%	45.00%

## Discussion

In recent years, more and more clinicians have begun to pay attention to the impact of cervical conization on pregnancy. Through a large sample size retrospective study, we identified that pregnancy after CKC was more likely to have adverse outcomes. Our study may help gain knowledge about the impact of CKC on pregnancy.

A history of previous cervical conization is one of the risk factors of cervical insufficiency [9]. It is generally considered that partial resection of cervical tissue weakens the mechanical support and extensibility of the cervix, and the proportion of collagen in the regenerated cervical tissue may be atypical of that of the original tissue, and the disproportionate collagen fiber ratio can lead to cervical insufficiency [10].

It is still controversial whether cervical cerclage is necessary for patients with fertility requirements after cervical conization. In our study, no significant difference in cervical cerclage rates between the two groups. The 2022 Royal College of Obstetricians and Gynaecologists (RCOG) guidelines list patients with a history of cervical conization as an intermediate-risk group for cervical insufficiency [9]. Some studies have shown that cervical cerclage effectively prolonged the cervical length [11]. Conversely, the study by Wang et al. [12] found that the incidences of preterm birth and premature rupture of membranes in the prophylactic cervical cerclage group were higher than those in the non-cerclage group. Presently, routine preventive cervical cerclage is not necessary for every patients after cervical conization. We can try to evaluate the possibility of cervical insufficiency according to the size of the patients’residual cervix. Patients with a history of miscarriage or premature delivery should be carefully evaluated and monitored, and cervical cerclage during pregnancy or before pregnancy should be considered.

In this study, the abortion rate after cervical conization was consistent with that of the normal population, considering that partial cervical resection may have reduced the cervix’s load-bearing capacity but could still tolerate the increased loading pressure of early and middle pregnancy. Spontaneous abortion may be affected by genetic, immune, endocrine, and other factors. Therefore, it is possible that cervical conization may not lead to a significant increase in abortion rate, but this cannot be confirmed as the results of secretion examination and cervical function evaluation of patients who aborted were not included in this study. However, a number of studies have suggested that cervical conization can lead to an increase in the rate of abortions [13,14]. They believed that the procedure reduces the cervical length and elasticity, weakens the compression capacity of the cervix, and reduces the mucus secretion of the cervical glands. Subsequently, a concomitant decrease in the secretion of lysosomal substances and immunoglobulin A, along with increased bacterial reproduction in the cervix and increased prostaglandin levels in the body, can result in early uterine contractions [15–17]. This mechanism accounts for the increase in preterm delivery and premature rupture of membranes in patients after conization [18,19].

The results of this study showed a higher incidence of preterm labor and premature rupture of membranes in pregnant women after CKC than in healthy pregnant women, and a higher percentage of premature rupture of membranes before term, which is one of the key problems causing preterm labor. Moreover, the repair after cervical conization can be equated to the process of inflammatory infiltration, which may change the immune microenvironment in the cervix, thus increasing the chance of infection [18]. Frega et al. [16] found that the most common pathogens of vaginal infection after cervical conization were Candida albicans, Gram positive vaginal bacteria, Group B Streptococci and Mycoplasma. It is further confirmed that persistent vaginal infection after cervical conization is one of the critical factors leading to adverse pregnancy outcomes. Some researchers believe that this may also be related to the scope of conization and the time interval of pregnancy after the operation. Some researchers have proposed that the depth of cervical conization >1 cm can significantly increase the premature delivery rate of patients. When the depth of conization is >2 cm, the risk of preterm delivery can be 5 times higher than that of the normal population [20]. In a study by Liverani et al. [21], patients with preterm deliveries had conical heights ≥15 mm or conization volume ≥2.0 cm^3^. Through correlation analysis, it was found that there was a significant negative correlation between cone taper height and gestational age (r = 0.3, P<0.001), but the transverse diameter and volume of conization were not related to gestational age. In this study, it was found that the transverse diameter of the cone floor had no significant effect on preterm delivery and premature rupture of membranes, but the cone height and volume may be related to the occurrence of preterm delivery and premature rupture of membranes. When the conical height is ≥25 mm, the risk of preterm delivery and premature rupture of membranes was eight times and 2.4 times higher than that of the normal population. When conical volume ≥4.0 cm^3^, the risk of preterm delivery and premature rupture of membranes was 9.5 times and 2.1 times higher than that of the normal population, respectively. Cone height is more consistent in predicting preterm delivery, while Sozen et al. [19] calculated that the critical values of cone height and cone volume for predicting preterm delivery were 2.25 cm and 2.27 cm3 respectively, while the critical values for predicting preterm premature rupture of membranes were 1.75 cm and 3.99 cm3, respectively. In addition, the study by Allah et al [22] did not suggest a difference in the rate of preterm delivery after conization compared to the normal population and calculated the mean height of cervical tissue removed in the conization group of 12.6 ± 5.4 mm and the mean length of the residual cervix after delivery of 28.7 ± 4.3 mm. Therefore, a few authors have suggested that there may be a threshold value for the effect of conization depth on pregnancy outcome. When the depth of conization exceeds a certain threshold value, it is associated with a higher risk of pregnancy outcome. The effect of the depth of conization on pregnancy outcomes may have a threshold. When the depth of conization exceeds a certain threshold, it is positively associated with some adverse pregnancy outcomes, while within a certain depth range, there is no significant effect. For example, Castanon et al [23] suggested that the incidence of preterm delivery is significantly increased only when the depth of resection is >1.5 cm or the volume of resection is >2.66 cm 3, but there is no definitive answer to this threshold. For patients who may desire fertility, clinicians should strictly avoid unnecessary tissue resection on the basis of ensuring the efficacy of treatment.

In terms of the time interval of postoperative pregnancy, this study found that the incidence of premature rupture of membranes within six months after conization was higher than after six months and more than 12 months after conization, which was consistent with the results of Conner et al. [24]. It may be related to the process of cervical repair after conization; that is, with the extension of time after the operation, the cervical wound and its surrounding tissue gradually recover. The cervix’s physical structure, secretory functions, and local immune microenvironment gradually recover over time, reducing the adverse outcomes of pregnancy. In contrast, some studies have shown that the adverse pregnancy outcomes caused by conization do not decrease with the prolongation of the pregnancy interval [23]. Rather, they claim that recovery time after cervical conization is related to the scope of surgical resection. The scope of conization depends on the location of cervical lesions, the type of transformation area, the number of conizations performed, and whether there is a clear desire to have children before the operation, so clinicians can monitor the pregnancy time of patients individually according to their condition perioperatively and postoperatively. Combining the results of most studies to date, it is recommended that patients should have at least 6 months of postoperative contraception. In addition, due to the limitations of retrospective studies in general, this study cannot compare preoperative cervical lengths. Our findings will be more conclusive if we can measure the cervical lengths of patients before surgery, at various points after surgery, and before and during pregnancy.

Besides cervix dysfunction, human papillomavirus (HPV) infection may also play a role in adverse pregnancy outcomes [25]. Some researchers believe that HPV-infected people often have an impaired immune barrier in the cervix, which is often complicated with other pathogenic infections, causing a pelvic inflammatory environment that is unfavorable for conception [26]. In addition, the HPV virus can bind to sperm heads and directly reduce sperm motility; the HPV virus attached to sperm and anti-HPV IgG antibodies in the cervix form a complex that can cause sperm agglutination and hinder fertilization [27]. In addition, a number of studies have shown that there are certain differences in the vaginal flora between patients with cervical lesions and the normal population, and considering the interaction between HPV infection and vaginal flora, and how cervical surgery can also cause changes in vaginal flora, which may affect conception and pregnancy stage, has an impact [28]. If the pre-pregnancy HPV infection of both husband and wife, the vaginal flora of the patient before and after surgery, and the pregnancy rate can be compared with the normal population, it will be more helpful to analyze the impact of cervical conization on pregnancy. In addition, some post-conectomy patients have a reduced libido, anxiety, and depression about the surgery and disease progression, which may affect conception in post-conectomy patients [29]. At the moment, we give natural ways of getting pregnant more weight for people who don’t have problems with fertility or infertility.

Increases in the rates of preterm delivery and premature rupture of membranes have apparent adverse effects on neonates [30]. In this study, the proportion of neonates admitted to neonatal pediatric units after their mothers underwent CKC was higher than that of the normal population. Even after eliminating the confounding factors surrounding preterm delivery, the incidence of neonatal intrauterine infectious pneumonia was still significantly higher compared to that in the normal population. A number of studies have confirmed that premature rupture of membranes can increase the risk of neonatal infection, which, in itself, is an independent risk factor for neonatal intrauterine infectious pneumonia. However, there is still a lack of research on intrauterine and neonatal infections in the population after conization. If the infection time, maternal and neonatal infection pathogen types, and prognosis can be compared to the normal population, it will be beneficial in guiding practitioners on perinatal and neonatal management after conization.

A history of cervical conization is not an indication for cesarean section, but a number of studies have shown that the rate of cesarean section in patients after conization is higher than that in the normal population, which is mainly affected by psychological factors [17,18]. The cesarean rate was higher in the CKC group in this study, and the number of non-medically indicated cesarean deliveries in the two groups was close to being statistically different (P = 0.051). The number of non-medically indicated cesarean sections is higher in the population after conization. The reasons for this increase include patients’ concerns about the influence of surgical history on vaginal delivery and some clinicians’ considering cervical scarring and large fetal size as medical indications for a cesarean section. The latter, or at the same time, caused the proportion of cervical laceration in patients after conization to be lower than that in normal people, and there was no case of cesarean section transferred to cesarean section because of "cervical dystocia". In recent years, most people tend to think that conization does not affect vaginal delivery. The results of Klaritsch et al. [31] show that there is no statistical difference in the mode of delivery, duration of labor, and induction rates between patients after conization and the normal population. Even though some studies have found that the risk of dystocia after conization increases [15], it does not prove that conization is the direct cause of dystocia. At present, there is a relative dearth of clinical research on the comparison of the duration of each stage of natural delivery after complication with that of the normal population. Clinicians should tell patients that the operation itself doesn’t change how the baby is born. This will help them relax and not worry about things that they don’t need to worry about. They should also actively encourage women who don’t have any reason not to have a vaginal delivery to try a vaginal trial of labor and closely watch the labor process.

In summary, the incidence of premature birth, premature rupture of membranes, and neonatal morbidity may be increased due to CKC for cervical lesions. Controlling the scope of resection and prolonging pregnancy intervals are crucial to avoid adverse pregnancy outcomes. Prospective studies with large sample sizes are needed to further determine the optimal management approaches for cervical lesion patients who have bearing desires.

## Supporting information

S1 File (DOCX)
