# Supplementary material for: The effect of cold-knife conization on pregnancy outcomes in patients with cervical lesions
Source: PLoS One. 2022 Dec 1;17(12):e0278505. doi: 10.1371/journal.pone.0278505 (PMC9714936; doi:10.1371/journal.pone.0278505)
Supplement: S1 File — (DOCX) [file pone.0278505.s001.docx]

**Tests for Two Proportions**

**Numeric Results for Testing Two Proportions using the Z-Test with Unpooled Variance**

H0: P1 - P2 = 0. H1: P1 - P2 = D1 ≠ 0.

**Target Actual Diff**

**Power Power* N1 N2 N P1 P2 D1 Alpha**

0.90 0.90090 125 125 250 0.3888 0.2050 0.1838 0.0500

* Power was computed using the normal approximation method.

**References**

Chow, S.C., Shao, J., and Wang, H. 2008. Sample Size Calculations in Clinical Research, Second Edition.

Chapman & Hall/CRC. Boca Raton, Florida.

D'Agostino, R.B., Chase, W., and Belanger, A. 1988. 'The Appropriateness of Some Common Procedures for Testing

the Equality of Two Independent Binomial Populations', The American Statistician, August 1988, Volume 42

Number 3, pages 198-202.

Fleiss, J. L., Levin, B., and Paik, M.C. 2003. Statistical Methods for Rates and Proportions. Third Edition.

John Wiley & Sons. New York.

Lachin, John M. 2000. Biostatistical Methods. John Wiley & Sons. New York.

Machin, D., Campbell, M., Fayers, P., and Pinol, A. 1997. Sample Size Tables for Clinical Studies, 2nd

Edition. Blackwell Science. Malden, Mass.

Ryan, Thomas P. 2013. Sample Size Determination and Power. John Wiley & Sons. Hoboken, New Jersey.

**Report Definitions**

Target Power is the desired power value (or values) entered in the procedure. Power is the probability of

rejecting a false null hypothesis.

Actual Power is the power obtained in this scenario. Because N1 and N2 are discrete, this value is often

(slightly) larger than the target power.

N1 and N2 are the number of items sampled from each population.

N is the total sample size, N1 + N2.

P1 is the proportion for Group 1 at which power and sample size calculations are made. This is the treatment

or experimental group.

P2 is the proportion for Group 2. This is the standard, reference, or control group.

D1 is the difference P1 - P2 assumed for power and sample size calculations.

Alpha is the probability of rejecting a true null hypothesis.

**Summary Statements**

Group sample sizes of 125 in group 1 and 125 in group 2 achieve 90.090% power to detect a

difference between the group proportions of 0.1838. The proportion in group 1 (the treatment

group) is assumed to be 0.2050 under the null hypothesis and 0.3888 under the alternative

hypothesis. The proportion in group 2 (the control group) is 0.2050. The test statistic used is

the two-sided Z-Test with unpooled variance. The significance level of the test is 0.0500.
